# Bridging Simulation and Sustainability: Laccase Immobilization on Bio-Polymeric Hybrids for Degradation of 17α-Ethinylestradiol in Water Systems

**DOI:** 10.1021/acsomega.5c13237

**Published:** 2026-04-20

**Authors:** Agnieszka Rybarczyk, Pranchal Shrivastava, Rukmankesh Mehra, Teofil Jesionowski, Anne S. Meyer, Jakub Zdarta

**Affiliations:** † Institute of Chemical Technology and Engineering, Faculty of Chemical Technology, 49632 Poznan University of Technology, Berdychowo 4, Poznan PL-60965, Poland; ‡ Department of Chemistry, 486388 Indian Institute of Technology Bhilai, Durg, Chhattisgarh 491002, India; § Department of Bioscience and Biomedical Engineering, Indian Institute of Technology Bhilai, Durg, Chhattisgarh 491002, India; ∥ Department of Biotechnology and Biomedicine, DTU Bioengineering, Technical University of Denmark, Soltofts Plads 227, Kgs, Lyngby DK 2800, Denmark

## Abstract

We present the development and comprehensive characterization of a biocatalytic system comprising laccase immobilized on a polystyrene-chitosan (PS-chitosan) carrier. The material was synthesized using electrospinning, which enabled the formation of a porous structure with a large surface area, offering favorable properties. Laccase from *Trametes versicolor* was successfully immobilized on the PS-chitosan with a yield of 87% and an activity retention of 83%. The immobilization was assessed by means of Fourier transform infrared spectroscopy (FTIR), confocal laser scanning microscopy (CLSM), electrokinetic potential measurements, and scanning electron microscopy (SEM). Kinetic analyses and thermodynamic studies confirmed that immobilization favorably influenced the catalytic properties of the laccase. Molecular docking and molecular dynamics simulations enabled identification of the preferred sites for binding of the enzyme to the material, as well as visualization of the interaction between the immobilized enzyme and the estrogen 17α-ethinylestradiol (EE2) substrate. The system enabled the removal of EE2 from model samples and real wastewater, achieving removal efficiencies of 86% and 44%, respectively. Overall, the study provides new insight into the immobilization binding of laccase to a PS-chitosan support and confirms the bioremediation potential of laccase immobilized on PS-chitosan, paving the way for developing efficient biocatalytic systems.

## Graphical abstract

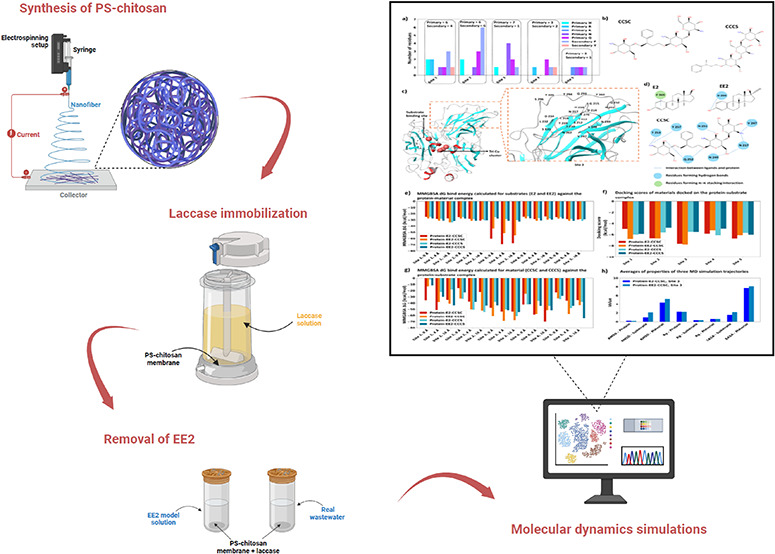

## 1 Introduction

An increasingly widespread environmental problem is the presence of hormonal agents in surface waters.^1^ Estrogens-natural or synthetic steroidal compounds, which in excessive amounts can lead to organ dysfunction and the development of many diseases-require particular attention.^2^ Biocatalytic systems are emerging as promising, environmentally friendly reactions to remove such compounds from wastewater.^3^ The main advantages of catalytic proteins include high selectivity and catalytic efficiency, enabling the removal of low concentrations of pollutants under mild process conditions.^4^ Particularly noteworthy are the laccase enzymes in the class of multicopper-oxidoreductases, which catalyze the oxidation of phenolic and aromatic (nonphenolic) compounds,^5^ and are thus particularly useful for bioremediation as well as other processes.^6,7^ Laccases are also of particular interest because the reaction uses O_2_ as cosubstrate and has water as its only byproduct; they are therefore increasingly used to remove phenolic pollutants from aquatic ecosystems.^8,9^ Unlike environmentally harmful synthetic oxidizing compounds, the use of laccases thus allows the enzymatic oxidation of pollutants to free radicals or quinones, which then undergo polymerization and partial precipitation, making the biocatalytic removal of pollutants an environmentally friendly process.^10^

However, the use of laccases is somewhat limited in practice by their low chemical or thermal stability and narrow pH range.^11^ A way to improve the stability and catalytic productivity of enzymes is their immobilization on supports, which not only increases the resistance of the biocatalysts to unfavorable environmental conditions, but also facilitates their recovery, providing the possibility of reuse.^12^ The efficiency of a process based on enzyme immobilization is impacted by the choice of immobilization technique, which determines the activity of the immobilized enzyme.^13^ Another important factor affecting the efficiency of biocatalysts after immobilization is the selection of an appropriate carrier, guided by knowledge of its chemical nature and mechanical properties.^14^ An extremely promising carrier is chitosan, whose natural origin ensures the carrier’s biocompatibility, biodegradability, and lack of toxicity.^15^ On the other hand, the effectiveness of immobilization depends on the content of free amino groups, which can be adjusted by changes in temperature, concentration, or time of exposure to the NaOH solution that causes deacetylation of chitin.^16^ The binding of enzymes to chitosan is possible via ionic or covalent bonds, due to the presence of amino groups in the structure of the carrier.^17^ However, determining the exact mechanism of binding of proteins to the carrier material is a challenge due to the complexity of the surface interactions.^18^ There are significant gaps in the literature concerning the occurrence of privileged binding sites in the enzyme structure, which can be identified using computer modeling to predict interactions between biocatalysts and the matrices on which they are deposited. This this paper therefore attempts to characterize these sites.^19^ Protein structure modeling is a state-of-the-art algorithm-based technique for predicting protein binding sites based on the AlphaFold and Protein Data Bank (PDB) databases.^20,21^ Konc et al.^22^ developed the ProBiS software, which proved to be a groundbreaking tool for predicting binding sites and appropriate ligands for a given protein structure. The developed algorithm has also been extended to several web servers, which enable rapid determination of binding sites for the entire PDB. Currently, it is one of very few such refined software tools, used primarily in the development of new drugs and vaccines.^22^

It would be an extremely innovative line of research to develop and understand the binding of the enzyme to the chitosan surface, in order to obtain new insight into the immobilized enzyme system. In this study, such research was carried out using laccase deposited on an electrospun PS-chitosan material. Besides detailed prediction of the enzyme’s binding to the support and the substrate’s binding to the enzyme, the report includes a detailed characterization of the newly formed polystyrene–chitosan material with immobilized enzyme, and the application of this biocatalytic system for estrogen removal.

## 2 Materials and Methods

### 2.1 Materials and Reagents

Polystyrene (PS), poly­(ethylene glycol)-*block*-poly­(propylene glycol)-*block*-poly­(ethylene glycol), *N*,*N*-dimethylformamide, acetic acid, chitosan, laccase from *Trametes versicolor* (EC 1.10.3.2), 2,2′-azino-bis­(3-ethylbenzothiazoline-6-sulfonic acid) (ABTS, 99%), and Bradford reagent were received from Sigma-Aldrich. 50 mM acetate buffer (pH 3–5), 50 mM phosphate buffer (pH 6–8), and 50 mM Tris-HCl (pH 8 and 9) were freshly prepared.

### 2.2 Synthesis of PS-Chitosan

In the first step, a chitosan solution was prepared by dissolving 0.04 g of chitosan in 3 mL of glacial acetic acid at 70 °C for 3 h. Next, a PS polymer solution was prepared by dissolving 0.96 g of polymer in 3 mL of DMF, followed by mixing for 24 h. After the PS was dissolved, two drops of PEG–PPG–PEG were added to its solution, serving as a surfactant in the system. After 3 h of mixing, 300 μL of the chitosan solution was added to the mixture. The resulting system was mixed for several more hours until a homogeneous solution was obtained. This solution was then placed in a syringe mounted on a KDS100 infusion pump, ensuring a constant flow rate of the mixture through the nozzle. To produce the electrospun material, the prepared solution was subjected to an electric field of 12 kV with a constant current of 1 mA. The distance between the collector and the needle was 10 cm, and the feed rate was 1 mL/h. The electrospinning process lasted 30 min. The obtained material was dried for 24 h at 27 °C in a Memmert dryer.

### 2.3 Laccase Immobilization

The immobilization of laccase from *Trametes versicolor* was performed on the synthesized PS-chitosan carrier. A protein solution with a concentration of 5 mg/mL was prepared by dissolving an accurately weighed amount of laccase in acetate buffer (pH 5). The immobilization process took place in glass vials, each containing 10 mg of carrier and 5 mL of the freshly prepared enzyme solution. The vials were agitated using an MS3 Digital mechanical shaker (IKA-Werke GmbH & Co. KG, Germany) at 150 rpm. The immobilization was carried out at ambient temperature for 4 h. After the immobilization step, the samples were washed three times with acetate buffer (pH 5) to remove unbound enzyme and subsequently stored in the wet state in the same buffer at 4 °C until use.

### 2.4 Structural and Physicochemical Characterization of the Produced Systems

The structural and physicochemical properties of the PS-chitosan systems before and after laccase immobilization were determined. FTIR spectra were obtained to identify the functional groups present in the samples. Measurements were carried out using a Vertex 70 spectrophotometer (Bruker, Germany) equipped with an attenuated total reflectance (ATR) attachment containing an embedded diamond, allowing total internal reflection of radiation. To analyze the distribution and localization of the immobilized enzyme, confocal laser scanning microscopy (CLSM) was performed using an Olympus Fluoview 500 microscope (Olympus, Japan). The acquired images were processed and analyzed with Fluoview (Olympus, Japan) and ImageTool 3.0 software (Department of Dental Diagnostic Science, University of Texas, USA). The surface charge of the PS-chitosan systems was determined using a SurPASS 3 electrokinetic analyzer (Anton Paar, Austria) by measuring the zeta potential. Prior to measurements, the samples were soaked in 1 mM KCl. The zeta potential was then recorded using 1 mM KCl as a background electrolyte over a pH range of 2 to 10, with pH adjustments performed via an automatic titrator. For each pH measurement point, four data points were recorded, and mean values were reported. The error margin for zeta potential measurements does not exceed 2%. The morphology of the PS-chitosan systems was examined using scanning electron microscopy (SEM). Images were obtained using an EVO40 microscope (Zeiss, Germany) after coating the samples with gold using a Balzers PV205P sputter coater (Switzerland). Conversely, the specific surface area was determined using the BET method based on nitrogen adsorption–desorption measurements conducted at low temperature over a range of decreasing relative pressures. The analyses were carried out using an ASAP 2020 instrument (Micromeritics Instruments Co., USA). Furthermore, pore size distribution and total pore volume were evaluated using the BJH method.

### 2.5 Biocatalytic Characterization of the Produced Systems

The catalytic activity of laccase, both free and immobilized on the PS-chitosan carrier, was evaluated using the model ABTS oxidation reaction at ambient temperature. For this purpose, 10 mg of the immobilized enzyme or an equivalent amount of free laccase was introduced into a reaction system containing 5 mL of a 15 mM ABTS solution prepared in 50 mM acetate buffer at pH 5. The equivalent amount of free laccase was calculated based on the experimentally determined enzyme loading of 2177.5 mg g^–1^ of carrier, meaning that 10 mg of the immobilized preparation corresponded to approximately 21.8 mg of free laccase. Prior to the activity assay, the immobilized enzyme samples were washed three times with fresh acetate buffer (pH 5) to remove unbound enzyme. The reaction mixtures were incubated under continuous mixing at 200 rpm for 10 min using a mechanical shaker, at ambient temperature and in the absence of light. The molar concentration of free laccase in the reaction mixture was approximately 0.078 mM, calculated using an average molecular weight of 64 kDa. Following incubation, absorbance was measured at 420 nm using a Jasco V-750 spectrophotometer (Japan). Absorbance was recorded continuously, and the enzymatic activity was calculated from the initial linear region of the absorbance–time curve, typically within the first 2–3 min of the reaction, during which the absorbance values remained within the linear detection range of the instrument. The activity recovery was determined by calculating the ratio of the activity of the immobilized enzyme to that of the free enzyme, expressed as a percentage, according to eq 1:

1 $$A=\frac{A_{1}}{A_{0}}\times 100\%$$

where *A* represents the activity recovery (%), *A*_1_ denotes the activity of the immobilized laccase, and *A*_0_ denotes the activity of the free enzyme.

To determine the quantity of laccase immobilized on the PS-chitosan carrier, the Bradford method was employed. The analysis was performed using styrene cuvettes, where 0.5 mL of the postimmobilization solution was mixed with an equal volume of Bradford reagent. After an incubation period of 5 min, absorbance was recorded at 595 nm using a Jasco V-750 spectrophotometer (Japan). A calibration curve was constructed based on bovine serum albumin (BSA) standards of known concentrations, enabling the determination of protein content. The amount of immobilized enzyme was calculated by assessing the difference in protein concentration before and after immobilization, relative to the mass of the carrier. The efficiency of immobilization (*I* %) on the carrier surface was determined by comparing the initial (*C*_*P*_) and residual (*C*_*K*_) protein contents in the solution, following eq 2):

2 $$I=\frac{C_{P}-C_{K}}{C_{P}}\times 100\%$$

To assess the impact of pH on the activity of both free and immobilized laccase, enzyme samples were incubated in 5 mL of a 15 mM ABTS solution prepared in different buffer systems: acetate buffer at pH 3, pH 4 and pH 5, phosphate buffer at pH 6, pH 7 and pH 8, and TRIS buffer at pH 9. The reactions were conducted at 25 °C for 10 min in darkness. Additionally, the effect of temperature on enzymatic activity was examined under similar conditions, with acetate buffer at pH 5 serving as a constant reaction medium while the ABTS oxidation reaction was performed at varying temperatures of 10, 20, 30, 40, 50, and 60 °C. For the determination of activation energy, initial reaction rates obtained in the temperature range of 30–60 °C were used. Arrhenius plots were constructed by plotting ln­(v) versus 1/T (K^–1^), and the activation energy was calculated from the slope of the linear regression according to the Arrhenius equation. In these tests, the highest recorded activity for each enzyme variant was defined as 100%.

Thermal stability was evaluated by incubating free and immobilized laccase at 50 °C in acetate buffer (pH 5) over a 180 min period. At predetermined time intervals, samples were collected for the ABTS reaction, with the initial activity of both enzyme forms being defined as 100%. The thermal inactivation constant (*k*_*D*_) was determined from the slope of the linear regression of ln­(residual activity) versus time, and the enzyme half-life (*t*__*1*_ */*_*2*__) was calculated accordingly. To determine storage stability, samples of free and immobilized laccase were stored at 4 °C for 30 days, with residual enzymatic activity measured at 5-day intervals. The maximum recorded activity in both cases was defined as 100%. Furthermore, the inactivation constant (*k*_*D*_) and enzyme half-life (*t*_1/2_) were calculated based on the slope obtained from linear regression analysis.

The oxidation reaction of ABTS under optimal conditions, with substrate concentrations ranging from 0.01 to 1 mM, was utilized to determine kinetic parameters, including the Michaelis constant (*K*_*m*_), maximum reaction rate (*V*_*max*_), and catalytic rate constant (*k*_*cat*_). Each experiment was conducted in triplicate, and the data obtained were analyzed using Hanes–Woolf plots to derive the kinetic parameters. The catalytic rate constant (*k*_*cat*_) was calculated according to the equation:

3 $$k_{\mathrm{cat}}=\frac{V_{\max}}{E_{t}}$$

where *V*_*max*_ denotes the maximum reaction rate (mol/s) and *E*_*t*_ is the total enzyme concentration (mol).

Furthermore, thermodynamic properties-enthalpy change (Δ*H*), entropy change (Δ*S*), and Gibbs free energy (Δ*G*)-were evaluated using the following equations:

4 $$\Delta H=E_{\mathrm{act}}-\mathrm{RT}$$

where *R* represents the gas constant (8.314 J/mol·K), *T* is the absolute temperature (K), and *E*_*act*_ denotes the activation energy (J/mol).

5 $$\Delta G=-\mathrm{RT}\ln\left(\frac{k_{\mathrm{cat}}\times h}{k_{b}\times T}\right)$$

where *h* is Planck’s constant (6.626 × 10^–34^ J·s), and *k*_*B*_ is Boltzmann’s constant (1.38 × 10^–23^ J·K^–1^).

6 ΔS=(ΔH−ΔG)/T

These thermodynamic parameters provide insights into the energetic and structural changes occurring during the catalytic process involving laccase immobilized on the PS-chitosan carrier.

### 2.6 Preparation of Protein, Substrates, and Materials

To understand the binding of the laccase to the material and substrate, we prepared structural models of the protein and ligands. The structure of laccase from *Trametes versicolor* was retrieved from the Protein Data Bank (PDB code 1GYC).^23^ The water molecules in the structure within 5 Å of Cu were retained. The protein structure was prepared at pH 5 using the Protein Preparation Wizard of Schrodinger.^24^

Two different models of the PS-chitosan material, comprising three units of chitosan (C) and one of polystyrene (S) (the same ratio was used to prepare the PS-chitosan material), were prepared and used: one with styrene at a terminal (labeled CCCS; Figure 4b) and the other with styrene between three chitosan molecules (CCSC; Figure 4b). The substrate, 17α-ethinylestradiol (EE2), and the carrier materials were prepared at pH 5 using ionizer in LigPrep.^25^

### 2.7 Binding Site Identification, Molecular Docking, and MMGBSA Analysis

The substrate binding site on laccase that lies near T1 Cu^2^ is well understood.^26–29^ For sampling substrate conformations, a 20 Å grid was generated around this site. EE2 was docked on this grid using the SP scoring function of Glide.^30^ A maximum of ten output poses were generated.

To determine the sites on laccase suitable for binding the material, we identified five potential binding cavities on the laccase–substrate (EE2) complex using SiteMap.^31^ The amino acid composition of these sites was analyzed and a 20 Å grid was generated on each of these sites in the presence of EE2 substrate. This led to a total of five grids (5 sites × 1 substrate–protein complex = 5 grids). The materials CCCS and CCSC were docked on these five grids, leading to ten complexes (5 grids × 2 materials = 10).

The ten complexes were subjected to Molecular Mechanics Generalized Born Surface Area (MMGBSA)^32^ analysis to identify the protein–ligand (substrate and material) binding affinities at three different flexibilities (0 Å, 4 Å, and 16 Å) of protein residues around the ligands. MMGBSA calculates the binding free energy as the difference between the energies of the minimized protein–substrate complex and minimized substrate plus minimized protein. The binding affinity of the substrates to the laccase–material complex was identified, and similarly the affinity of the material to the protein–substrate complex was determined. Therefore, a total of 60 protein–ligand binding affinities were evaluated (10 complexes × 3 flexibilities × 2 material/substrate = 60 affinity evaluations).

### 2.8 Molecular Dynamics Simulations

The combination of the protein with EE2 bound at the substrate binding site, along with the material CCSC, was selected for molecular dynamics studies, as this combination led to higher binding affinity in prior analyses (at site 3). These systems were prepared within rectangular simulation boxes containing TIP3P water molecules, neutralized with counterions, and accompanied by 0.15 M NaCl.^33^ Molecular dynamics (MD) simulations were conducted using the leapfrog algorithm to integrate Newton’s equations of motion, with a maximum of 250,000,000 steps and a 2 fs time step. Temperature coupling was achieved via velocity rescaling with a stochastic term, while pressure coupling was managed using exponential relaxation to ensure accurate volume fluctuations.^34^ Pressure and temperature were maintained using the C-rescale barostat (a modified Berendsen barostat) and the V-rescale thermostat, respectively.^34,35^ Periodic boundary conditions were applied in all directions, and the Verlet cutoff scheme was utilized during the minimization, equilibration, and production phases.^36,37^

Three independent MD simulations of each system were performed, each lasting 500 ns (500 ns × 3 runs × 1 system), yielding a cumulative simulation time of 1.5 μs across all systems. Random velocity seeds were assigned for each independent run to enhance statistical reliability. Simulations were conducted with the CHARMM36 force field using GROMACS 2023.^38–41^

### 2.9 Removal of Estrogen

To evaluate the potential use of the developed catalytic systems for the biodegradation of environmental micropollutants, the process of removal of 17α-ethinylestradiol (EE2) was conducted using both model solutions and real wastewater samples collected from a wastewater treatment plant located on the outskirts of Poznan. The efficiency of degradation of EE2 by immobilized laccase or the corresponding amount of free enzyme was examined as a function of process duration. Samples were prepared by adding 10 mL of model EE2 solution (concentration 300 ng/L) or real wastewater (pH 7.2; 352 ng/L EE2) to a carrier containing 10 mg of laccase or a proportional amount of free biocatalyst. The systems were mixed at 150 rpm using a shaker at ambient temperature, and the degradation process was carried out for 1, 2, 4, 6, 10, 12, and 24 h. At specified time intervals, 1 mL of solution was transferred from each catalytic system to an Eppendorf tube and analyzed using GC-MS to obtain the necessary data for calculating the removal efficiency (%), which represented the difference between the initial and final estrogen concentration in the solution. For gas chromatography analysis, 0.5 mL of the postdegradation samples was dried for 24 h at 45 °C using an Eppendorf Concentrator Plus (Darmstadt, Germany). Afterward, derivatization was carried out by adding 0.1 mL of a BSTFA + 1% TMCS reagent. The samples were subsequently analyzed on a Pegasus 4D GCxGC-TOFMS system (Leco Corp., St. Joseph, MI, USA) fitted with a BPX5 chromatographic column (30 m × 250 μm × 0.25 μm) produced by SGE Analytical Science Europe Ltd. (Milton Keynes, UK). The concentrations of EE2 were determined using calibration curves established from a series of standard solutions in wastewater with concentrations ranging from 10 to 1000 ppm, enabling the calculation of the EE2 levels in the experimental samples. The degradation efficiency (*D*_*e*_) was then calculated according to the following formula:

7 $$D_{e}=\left(\frac{C_{0}-C_{m}}{C_{0}}\right)\times 100\%$$

where *C*_0_ represents the initial concentration of EE2 and *C*_*m*_ is the concentration measured after enzymatic biotransformation.

In the final stage of the study, the possibility of reusing the biocatalytic systems over five consecutive 24-h reaction cycles was also investigated. For this purpose, 10 mL of the model or real solution and the immobilized laccase carrier were placed in glass vials. The degradation process was conducted at room temperature under continuous shaking of the prepared systems. After each reaction cycle, the immobilized system was washed with acetate buffer (pH 5) and transferred into fresh real solution.

The acute toxicity of 17α-ethynyloestradiol before degradation and of samples containing its degradation products was evaluated using the *Artemia salina* bioassay. *Artemia salina* cysts (0.5 g) were hatched in 500 mL of NaCl solution (25 g/L) at 25 °C under continuous illumination for 24 h. Subsequently, ten larvae were exposed to test solutions for 24 h at 25 °C. After exposure, mortality was recorded, and the percentage of dead larvae was calculated. All experiments were performed in triplicate.

### 2.10 Examination of Potential EE2 Degradation Pathways

Metabolite analysis was performed using an LC-MS system consisting of a 1290 Infinity II LC System (Agilent Technologies, Santa Clara, USA) coupled with a Q-TOF 6546 mass spectrometer (Agilent Technologies, Santa Clara, USA). The separation of analytes was achieved using a ZORBAX RRHD Eclipse Plus C18 column (95 Å, 1.8 μm, 2.1 × 50 mm, Agilent Technologies, Santa Clara, USA), maintained at a temperature of 25 °C. The mobile phase consisted of 1 mM ammonium formate in water (A) and acetonitrile (B), with a 0.5 mL/min flow rate. The following gradient was applied: 0 min: 30% B, 5 min: 95% B, 5–7 min: 95% B, 7–10 min: 30% B. The injection volume for each sample was 20 μL. Detection was performed in scan mode with negative ionization using a Dual AJS ESI ion source (Agilent Technologies, Santa Clara, USA). The following ion source parameters were applied in the mass spectrometer: drying gas temperature 300 °C; drying gas flow rate 10 L/min; nebulizer pressure 45 psi; sheath gas temperature 350 °C; sheath gas flow rate 11 L/min; capillary entrance voltage 3000 V; nozzle voltage 1000 V.

Total organic carbon (TOC) was measured according to Merck procedure no. 1.14879.0001, range: 50–800 C/L (Merck Millipore, Billerica, MA, USA). Samples were diluted with demineralized water at a ratio of 1:10, and the TOC concentration was measured with a photometer.

## 3 Results and Discussion

### 3.1 Properties of PS-Chitosan Material before and after Laccase Immobilization

For a comprehensive determination of the morphological and physicochemical properties of the electrospun PS-chitosan material before and after laccase immobilization, Fourier transform infrared spectroscopy (FTIR), confocal microscopy (CLSM), zeta potential measurement, and scanning electron microscopy (SEM) were applied (see Figure 1). These results provide detailed information on the structural changes and surface properties, and suggest a mechanism for the binding of the enzyme to the support.

**Figure 1:**
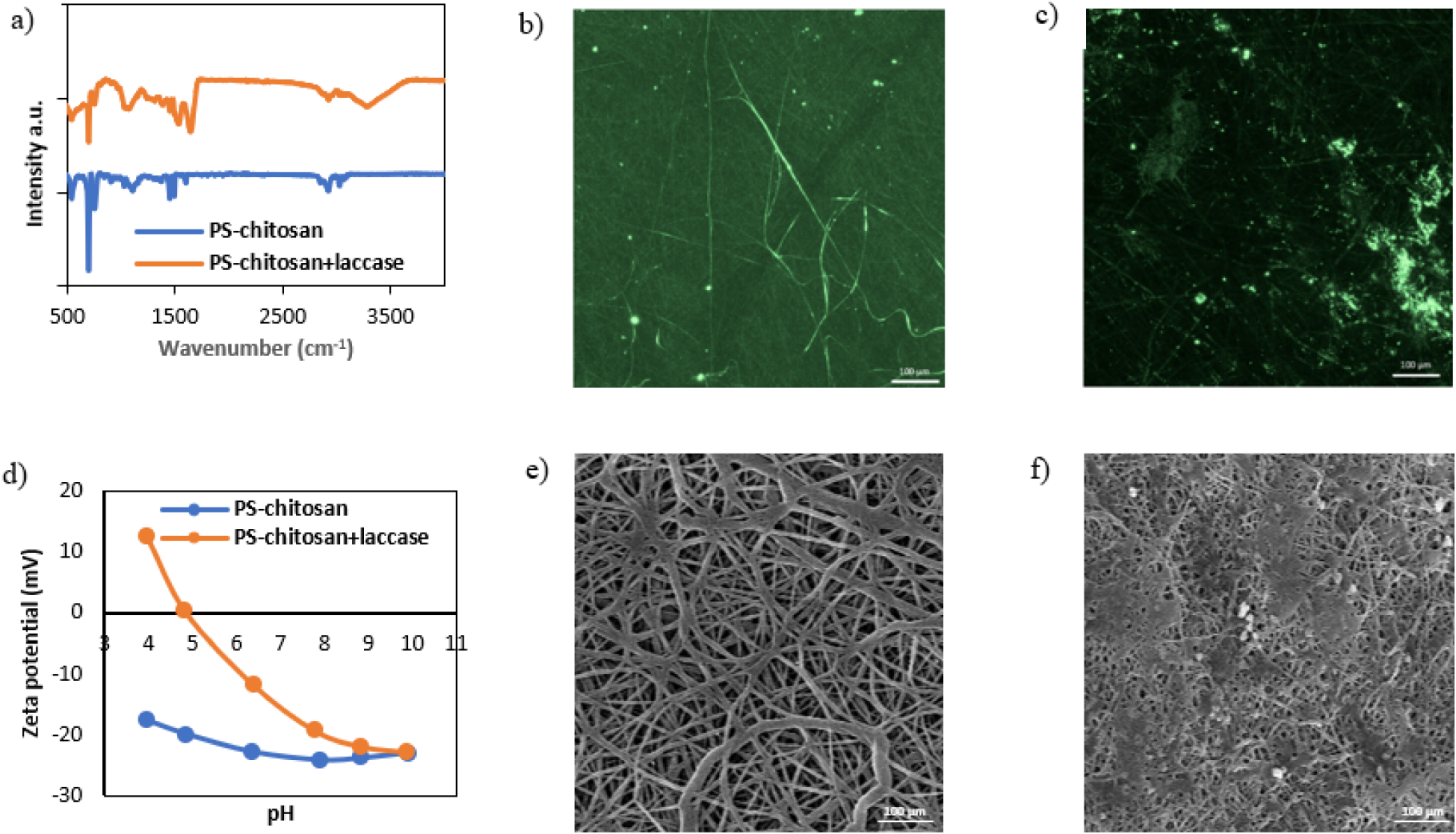
Morphological characterization ((b, c) CLSM and (e, f) SEM photos) and physicochemical characterization ((a) FTIR, (d) zeta potential) of the PS-chitosan material before and after immobilization.

FTIR is a method commonly used to analyze the chemical structure of materials. It enables the identification of specific functional groups based on characteristic vibrational modes, making it a standard technique for studying polymeric and biopolymeric systems. The FTIR spectrum of the PS-chitosan contains several significant absorption bands. The band in the 3000–3200 cm^–1^ range is related to the stretching of hydroxyl (−OH) and amine (−NH_2_) groups, typical of chitosan-based materials.^42^ In the lower ranges, around 1640 cm^–1^, a band attributed to the carbonyl groups in the chitosan structure can be observed. Additional bands with maxima slightly below 3000 cm^–1^ and in the 700–800 cm^–1^ region may be related respectively to the stretching and deformation vibrations of CH_3_ and CH_2_ groups.^43^ Such aliphatic contributions are consistent with polysaccharide backbones and are typically used as spectral markers in similar biomaterials. Significant changes are observed in the spectrum of PS-chitosan+laccase compared with that of the original material. The appearance of more intense bands in the 1650–1550 cm^–1^ range can be attributed to the presence of amide groups I and II, which are characteristic of protein structures such as laccase.^44^ Amide I (mainly C=O stretching) and amide II (N–H bending coupled with C–N stretching) vibrations are widely used to confirm the incorporation of proteinaceous species into polymer matrices and may additionally reflect elements of the enzyme’s secondary structure. The observed bands indicate the formation of mainly adsorption interactions between the enzyme and the carrier, confirming the effective immobilization of the enzyme. The presence of characteristic amide bands indicates interactions between the enzyme and the carrier, which may result mainly from hydrogen bonding and electrostatic forces between the functional groups of chitosan and laccase. These mechanisms are consistent with reported immobilization pathways for laccase on chitosan, where protonated amino groups and hydroxyl functionalities facilitate noncovalent binding. Additionally, under the applied experimental conditions, enzyme immobilization is primarily governed by noncovalent interactions. These include hydrogen bonding and electrostatic forces between the amino and hydroxyl groups of chitosan and charged or polar residues of the enzyme. The presence of a signal with a maximum around 3300 cm^–1^, characteristic of O–H and N–H stretching vibrations in amino acids, confirms the incorporation of protein onto the carrier surface. While FTIR analysis alone does not provide definitive evidence for covalent bond formation, the observed shifts and intensity variations in characteristic absorption bands suggest changes in the local chemical environment resulting from enzyme–support interactions. Possible covalent linkages between chitosan amino groups and enzyme carbonyl functionalities cannot be entirely excluded; however, such reactions are expected to be rare in the absence of surface activation or cross-linking agents and are therefore not considered the dominant immobilization mechanism in this system. These structural interactions contribute to the enhanced stability and activity of the immobilized enzyme in biotechnological applications. The differences observed between the spectra of PS-chitosan, free laccase, and PS-chitosan+laccase support the successful attachment of laccase to the carrier surface.

Analysis of the images obtained by confocal laser scanning microscopy (CLSM) allows a detailed evaluation of the distribution of laccase on the surface of the PS-chitosan material and a qualitative verification of the efficiency of enzyme immobilization. In the image representing a PS-chitosan sample without enzyme (Figure 1b), no fluorescence signal was observed, indicating the purity of the support material and the absence of any additional substances such as proteins or other fluorescent components. By contrast, in the PS-chitosan+laccase sample (Figure 1c), an intense and clear fluorescence signal was observed, clearly indicating the presence of the enzyme on the surface of the carrier. The fluorescence is the result of effective laccase binding to PS-chitosan. The lack of homogeneity of the fluorescence signal over the entire carrier surface suggests that the enzyme is not uniformly distributed. Noticeable brighter areas in the fluorescence image indicate locally higher enzyme loading. These more intense areas may be the result of preferential binding of the enzyme to areas of higher chemical reactivity, for example where there are more functional groups capable of interacting with the laccase. They may also be related to the surface topography of the PS-chitosan, where microscopic irregularities or specific structures may have increased local adsorption of the enzyme.

In turn, the zeta potential is a key parameter that characterizes the electrokinetic properties of materials as a function of pH. Analysis of this parameter allows a detailed understanding of the changes in electrostatic properties that occur on the surface of the PS-chitosan material after laccase immobilization, which in turn affect colloidal stability and intermolecular interactions. In the case of PS-chitosan (Figure 1d), negative zeta potential values are observed over a wide pH range from 4 to 10. This behavior can be attributed to the presence of amino groups derived from chitosan, which are capable of protonation in acidic media and deprotonation in basic media, which also forces the creation of electrostatic interactions between the enzyme and the support. After the immobilization of laccase, the zeta potential becomes significantly less negative over the analyzed pH range. This change is due to the deposition of enzyme molecules on the surface of the support, introducing new functional groups within the protein structure. Amino acids have isoelectric points (IEPs) which vary depending on their structure and functional groups, and they influence the electrostatic character of the modified surface. Laccase, depending on its source, typically has an isoelectric point in the range 3–5, suggesting a higher content of acidic amino acids such as aspartic acid (pI ≈ 2.8) and glutamic acid (pI ≈ 3.2), which contribute negative charges at neutral pH. In contrast, basic residues such as lysine (pI ≈ 9.7), arginine (pI ≈ 10.8), and histidine (pI ≈ 7.6) may locally influence the charge distribution, especially at catalytic sites.^6^ The presence of carboxyl (−COOH) and amino (−NH_2_) groups in the protein structure affects the value of the zeta potential, depending on their protonation state. The isoelectric point of the system at pH 5, similar to the pI of laccase, observed after laccase immobilization, further suggests modifications in the surface charge distribution, likely resulting from the incorporation of protein functional groups.

SEM is another extremely valuable tool for obtaining detailed information on the support morphology changes that occur as a result of enzyme immobilization. In the image of PS-chitosan before immobilization (Figure 1e), a characteristic network of irregularly arranged fibers can be observed, forming a highly porous structure. This type of morphology is typical of electrospun chitosan-based materials, and results from their formation process and the characteristic properties of the polymer, such as its ability to form a three-dimensional structure. The porosity of this structure provides a large surface area, which is essential for efficient enzyme immobilization. The presence of numerous pores and irregularly distributed fibers suggests the possibility of diffusion of substrate molecules and enzyme reaction products, which increases the efficiency of such a system. The fibers in this structure have diameters in the range 5–10 μm, while the interfiber external pores reach sizes of 10–60 μm, further facilitating mass transport. Additionally, apart from these macropores, the fibers themselves contain micropores, which enhance the diffusion of reactants and products within the structure, improving catalytic efficiency. In the morphology of the material after the immobilization process (Figure 1f) significant changes are visible, as enzyme molecules can be observed on the surface of the fibers. These deposits appear irregular and locally differentiated, indicating effective binding of laccase to the chitosan surface. The change in the surface structure, as evidenced by the increased thickness of the fibers and the irregularity of their edges, confirms the presence of the enzyme immobilized on the material. Moreover, the partial reduction in the size of large pores suggests that the immobilization process has altered the overall porosity of the system. The observed modifications in fiber structure and porosity further support the enhancement of mass transport within the system, optimizing conditions for enzymatic reactions. The electrospun PS-chitosan material thus offers a highly favorable environment for enzyme immobilization, combining large surface area, high porosity, and biocompatibility. To complement SEM observations, BET surface area and BJH pore size distribution analyses were performed. The PS–chitosan carrier exhibited a specific surface area of 18.7 m^2^/g and an average pore diameter of approximately 2.4 nm, confirming the presence of a mesoporous structure. Such porosity is advantageous for enzyme immobilization, as it increases the available contact area and may facilitate diffusion of substrates and products within the carrier matrix. However, one of the main limitations of chitosan is its poor mechanical strength, which can restrict its possible applications. To overcome this drawback, various strategies have been proposed. For example, chemical cross-linking with glutaraldehyde can enhance the rigidity of the fibers, while combining chitosan with more mechanically robust polymers during electrospinning improves structural integrity.^45,46^ Notably, Matsuda et al.^47^ developed a method to significantly enhance the tensile strength and elasticity of chitosan fibers by coating them with a secondary chitosan layer of variable thickness. This resulted in a material with high entropic elasticity and improved resistance to mechanical deformation, which is especially beneficial for reusable or flow-through biocatalytic systems.^47^

### 3.2 Characterization of the Laccase Enzyme before and after Immobilization

The immobilization of enzymes on supports such as PS-chitosan is a key step in biotechnological processes, allowing increased enzyme stability, the possibility of reuse, and optimized performance in industrial applications. Table 1 provides a detailed characterization of the laccase immobilization process, including enzyme activity recovery, immobilization yield, amount of immobilized enzyme, kinetic parameters (*K*_*m*_ and *V*_*max*_), and enzyme elution rate. These parameters provide a comprehensive evaluation of the immobilization efficiency and its impact on enzymatic performance, enabling the assessment of structural and functional stability, catalytic efficiency, and the potential applicability of the immobilized system in industrial processes.

**Table 1:** Characterization of the Immobilization Process, Enzyme Loading on PS-Chitosan Material, and Activity and Kinetic Parameters of the Free and Immobilized Laccase^a^

	Free laccase	SD	Immobilized laccase	SD
Activity recovery	100%	-	83.4%	2.6%
Immobilization yield	-	-	87.1%	3.1%
Amount of immobilized enzyme	-	-	2177.5 mg/g	13.4 mg/g
Enzyme loading	-	-	87.1 mg/cm^2^	4.2 mg/cm^2^
Enzyme leaching	-	-	26.1%	3.2%
*K*_*m*_	0.74 mM	0.03 mM	0.89 mM	0.04 mM
*V*_*max*_	0.119 mM/s	0.013 mM/s	0.082 mM/s	0.007 mM/s
*k*_*cat*_	1.523 1/s	0.013 1/s	1.049 1/s	0.007 1/s

a Results are presented as mean ± standard deviation of three replicate experiments.

The activity recovery of the immobilized laccase was 83.4%, indicating that a significant part of the enzymatic activity was retained after immobilization. Despite a partial decrease in activity compared to the free enzyme, this result is favorable from a practical point of view, considering the improved stability of the enzyme as a result of its immobilization. Almost all of the enzyme used in the process was effectively bound to the carrier, as the immobilization yield was calculated at 87%. The high efficiency of this process highlights the ability of PS-chitosan to bind enzymes, due to its large active surface area and the presence of chemical functional groups favoring enzyme immobilization and stabilization. The amount of immobilized laccase was 2177.5 mg/g of carrier, demonstrating the high capacity of PS-chitosan as an enzyme immobilization material. The efficiency of the process of coating the carrier with the enzyme was also confirmed by the enzyme loading value of the surface of the ossifying carrier (87 mg/cm^2^). The leaching rate of the enzyme from the carrier was determined to be 26%, which indicates that some laccase may have desorbed under experimental conditions. This result indicates a moderate stability of enzyme binding to the carrier, which is characteristic of adsorption immobilization, where enzymes interact with the support primarily through noncovalent forces such as hydrogen bonds, van der Waals interactions, and electrostatic interactions. The observed enzyme leaching rate of 26% suggests that while a significant portion of the enzyme remains attached, some molecules may desorb due to the reversible nature of these interactions, distinguishing this method from covalent immobilization, which typically ensures stronger and more permanent enzyme attachment, but requires more complex technological preparation.

The analysis of the enzyme’s kinetic parameters showed that the *K*_*m*_ value for free laccase was 0.74 mM, while for immobilized laccase it increased to 0.89 mM. The increase in *K*_*m*_ after immobilization is a well-known property of immobilized enzymes, which may be due to local diffusion limitations that decrease the substrate diffusion relative to the enzymatic rate. However, the increase in the apparent *K*_*m*_ from 0.74 mM to 0.89 mM indicates very limited substrate diffusion effects in the system. Likewise, the maximum reaction rate for laccase after immobilization decreased from 0.119 mM/s to 0.082 mM/s. This decrease may also be attributed to mass transport effects after immobilization, given that the actual substrate concentration near the immobilized enzymes is lower than in the bulk liquid (the enzymatic rate exceeds the substrate supply rate due to low substrate diffusion at the surface and within the porous immobilization material). Additionally, *k*_*cat*_ slightly decreased upon immobilization: from 1.523 s^–1^ for free laccase to 1.049 s^–1^ for immobilized laccase, a reduction of about 30%. While this change is noticeable, it remains within typical limits for such enzymatic systems and does not indicate a significant loss of catalytic activity. The changes in kinetic parameters of the laccase upon immobilization may be considered insignificant (around 20%), especially when considered alongside the retention of the catalytic robustness of the immobilized laccase.

Table 2 summarizes the thermodynamic and stability parameters for free and immobilized laccase, allowing a detailed comparison between the two forms of the enzyme in terms of energy activation, enthalpy and entropy changes, inactivation constant, and half-life. The activation energy (*E*_*a*_), which determines the amount of energy required to initiate an enzymatic reaction, is significantly lower for free laccase (31.62 kJ, against 55.96 kJ for the immobilized enzyme). The higher activation energy for immobilized laccase suggests that the catalytic process requires a higher energy input, which may be related to diffusion limitations or conformational changes of the enzyme due to its binding to the PS-chitosan carrier. Similar observations were reported by Lin et al.,^48^ who found that the activation energy increased from 29.92 to 66.39 kJ/mol after laccase immobilization.^48^ Nevertheless, immobilization leads to improved stability of the enzyme, as confirmed by other thermodynamic parameters. The Gibbs free energy (Δ*G*) is a measure of the overall energy difference between reactants and products of the catalytic process. The values of Δ*G* for the free laccase and the immobilized laccase were similar (87 kJ and 82.07 kJ, respectively), indicating that the laccase-catalyzed process (reaction trajectory) remained the same after immobilization. Interestingly, the enthalpy change (Δ*H*), a measure of the total energy exchanged during catalysis, is lower for the immobilized laccase (29.14 kJ) than for the free enzyme (53.48 kJ). The lower Δ*H* value indicates a lower energy cost when the structure of the biocatalyst is stabilized through immobilization, confirming the positive effect of immobilization on enzyme efficiency and stability.

**Table 2:** Thermodynamic and Stability Parameters of the Free and Immobilized Laccase^a^

	Free laccase	SD	Immobilized laccase	SD
Activation energy (*E*_*a*_)	31.62 kJ	-	55.96 kJ	-
Gibb’s free energy (Δ*G*)	87.56 kJ	-	82.07 kJ	-
Enthalpy change (Δ*H*)	53.48 kJ	-	29.14 kJ	-
Entropy change (Δ*S*)	–114.4 J	-	–177.6 J	-
Inactivation constant (*k*_*D*_)	0.0119 1/min	0.003 1/min	0.0011 1/min	0.0004 1/min
Enzyme half-life (*t*_1/2_)	58.5 min	4.2 min	632.7 min	12.3 min

a Results are presented as mean ± standard deviation of three replicate experiments.

The change in entropy (Δ*S*), which reflects the degree of order in the system during catalysis, is also an important parameter and shows significant differences between the two forms of the enzyme. The value of Δ*S* for free laccase is −114.4 J, while for the immobilized form it drops to −177.6 J. The decrease in entropy in the case of immobilized laccase suggests a greater orderliness of the reaction system and limited freedom of movement of the enzyme, which is exactly the expected effect of enzyme immobilization on the support. In a study by Wehaidy et al., a lower ΔS value for laccase immobilized on nanoporous zeolite-X compared to the free enzyme was reported, indicating a decrease in entropy during transition state formation, which stabilizes the enzyme–substrate complex and makes it more ordered.^49^ Such an effect may lead to stabilization of the enzyme structure, which is consistent with the observed persistence results. Another important parameter confirming the improvement of enzyme durability upon immobilization is the inactivation constant (*k*_*D*_), which describes the rate of degradation of enzyme activity. Free laccase has a higher inactivation constant of 0.012 min^–1^, while for immobilized laccase the value is only 0.0011 min^–1^. The significant decrease in the *k*_*D*_ value for the immobilized enzyme signifies a much slower inactivation rate, which is a manifestation of the stabilizing effect of the enzyme’s immobilization on the PS-chitosan carrier. A final confirmation of the improved stability of the enzyme as a result of immobilization is the enzyme half-life (*t*_1/2_), which increases more than 10-fold, from 58.5 min for free laccase to 632.7 min for immobilized laccase. This considerable increase in half-life reflects the increased robustness of the enzyme as a result of immobilization on PS-chitosan. As discussed below, the thermal stability of the enzyme also improved upon immobilization.

The stability and activity of enzymes under different pH and temperature conditions are also important for their practical application in biotechnological processes. In the present study, the effects of pH and temperature on the relative activity of laccase in free form and immobilized on the PS-chitosan support were investigated using a model solution to determine the most suitable operational conditions (Figure 2).

**Figure 2:**
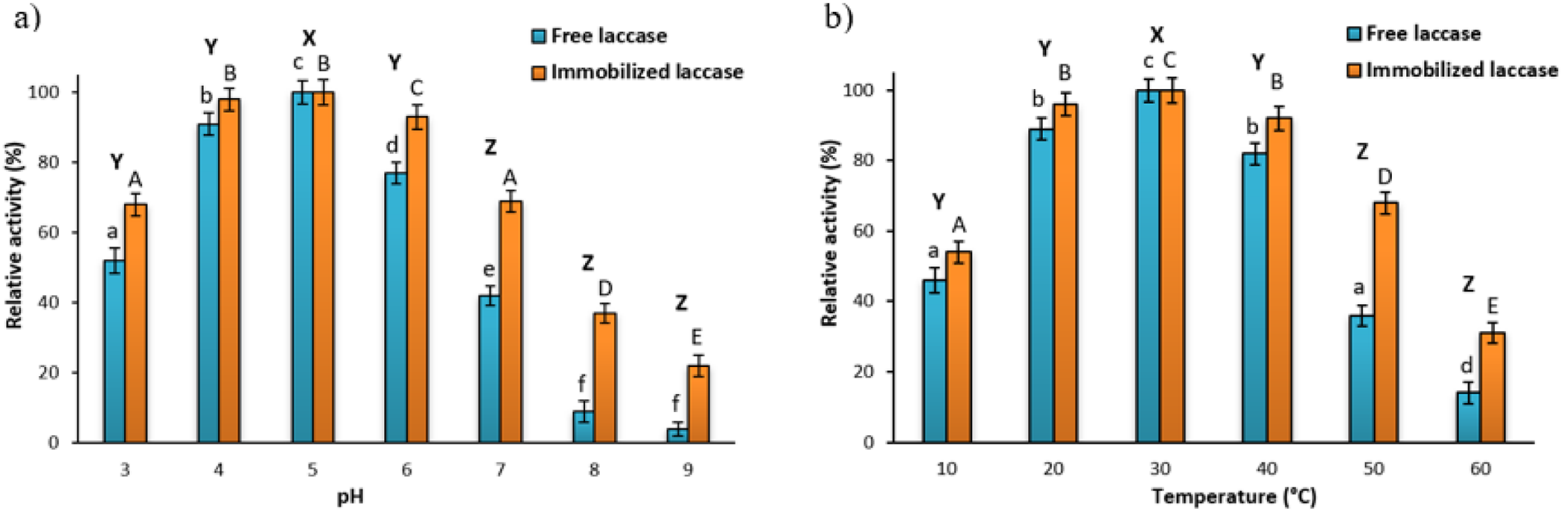
Effect of pH (a) and temperature (b) on the relative activity of the free and PS-chitosan immobilized laccase. Results are presented as mean ± standard deviation of three replicate experiments. The letters a, b, c, d, e, f represent significant differences among free laccase, while A, B, C, D, E represent significant differences between immobilized laccase, and X, Y, Z represent significant differences between relative activity. Means with different letters (a, b, c, d, e, f and A, B, C, D, E and X, Y, Z) are significantly different (ANOVA, *p* < 0.05).

Analyzing the effect of pH on the relative activity of free and immobilized laccase (Figure 2), it is found that for both free and immobilized laccase, it reaches its maximum at pH 5. While the immobilized enzyme also showed optimum activity at pH 5, it maintained a higher activity in a more alkaline environment compared with the free form, which lost almost all activity at pH 8 and 9. These results indicate that immobilization of the enzyme on PS-chitosan increases its resistance to pH changes, which may be related to the protective effect of the support, stabilizing the enzyme structure. Nevertheless, the pH curves for free and immobilized laccase showed a similar trend, indicating that no significant changes, besides enzyme stabilization, occurred in the enzyme microenvironment upon binding. In turn, analysis of the effect of temperature (Figure 2b) shows that the activity of free laccase increases with increasing temperature, reaching a maximum at 30 °C, after which a sharp decrease in activity is observed, particularly at temperatures above 50 °C. In the case of immobilized laccase, the temperature optimum also falls in the 20–30 °C range, but the enzyme retains significantly higher activity over a wider temperature range. This phenomenon is indicative of the increased thermal stability of the immobilized enzyme, maybe due to a reduction in protein denaturation as a result of its interaction with the PS-chitosan carrier, which might also act as a heat absorber and as a protective shield against thermal dissociation of enzyme subunits. Similar results were obtained in a study by Mazlan and Hanifah,^50^ who immobilized laccase on microspheres of a copolymer of glycidyl methacrylate and n-butyl acrylate (poly­(GMA-co-nBA)). In their work, the optimum pH for the free laccase was found to be 4, while for the immobilized laccase, it shifted to pH 5. Moreover, the immobilized laccase retained its activity over a wider range of pH and temperature values compared with the free form of the enzyme.

Analysis of the stability of enzymes is also an extremely significant step in assessing their application potential. In the vast majority of cases, the immobilization of enzymes leads to an increase in their thermal stability and half-life, which is important for their practical use. In the present study, the stability of laccase in free form and immobilized on a PS-chitosan carrier was compared by analyzing its thermal stability and storage stability (Figure 3).

**Figure 3:**
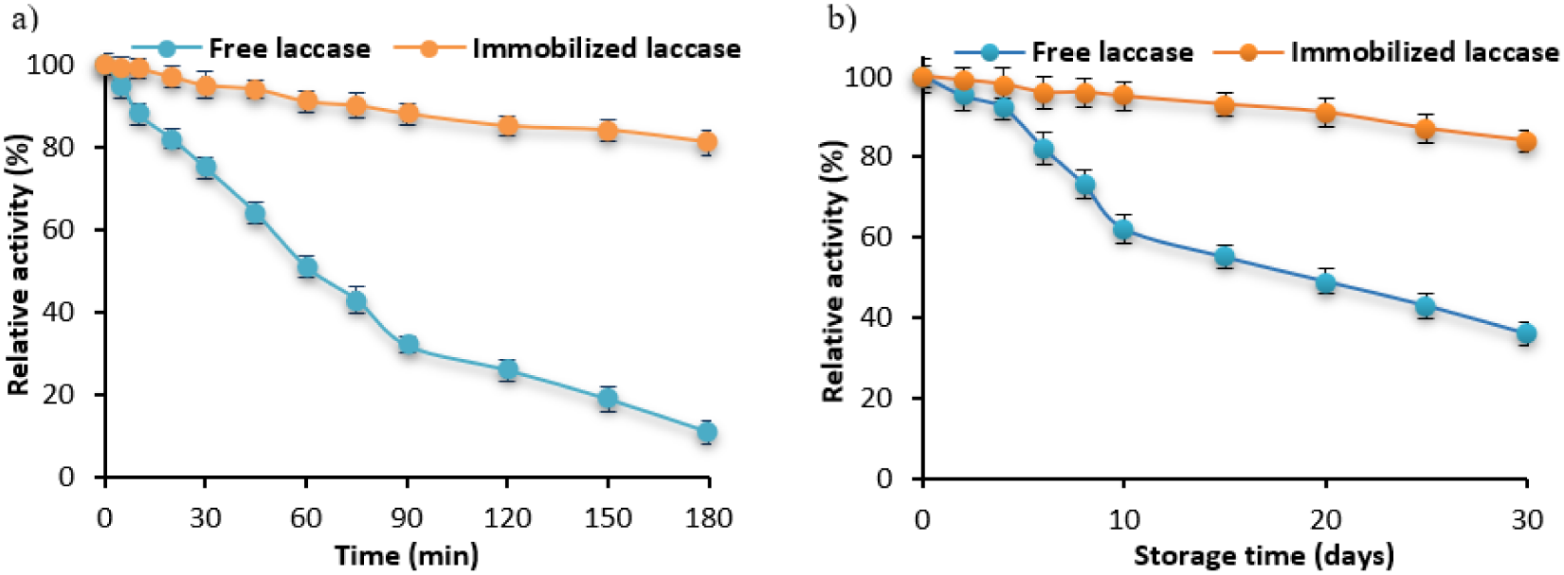
Thermal stability (a) storage stability (b) of the free and PS-chitosan immobilized laccase.

For the thermal stability analysis (Figure 3a), enzyme activity was monitored during incubation at 50 °C for 180 min. Free laccase showed a sharp decrease in activity within the first few minutes of incubation, losing almost 50% of its initial activity after 60 min and reaching a relative activity of about 10% after 180 min. This significant decrease can be attributed to the denaturation of the enzyme at high temperatures, leading to irreversible changes in its structure. In contrast, the immobilized laccase had a much higher stability, retaining about 80% of its initial activity after 180 min. The high stability of the immobilized enzyme is due to the protective effect of the PS-chitosan carrier, which stabilizes its structure and prevents denaturation, minimizing the negative effects of high temperature. Storage stability analysis (Figure 3b) showed similar trends over the longer term. During the 30-day storage period, the activity of free laccase gradually decreased to an apparent value of about 35% of the initial activity. This significant loss of activity may be related to the degradation of the enzyme due to various processes destabilizing its structure. In contrast to the free enzyme, the immobilized laccase retained much higher activity, suggesting that the PS-chitosan carrier plays a protective role, preventing degradation of the enzyme and providing greater durability in long-term storage.

### 3.3 Docking, Binding Affinity, and Molecular Dynamics Simulation Results

A detailed computational analysis was performed to understand the binding of the enzyme to the material, and to assess the mode of binding of the substrate to *T. versicolor* laccase (Figure 4). Experimental evidence suggested that laccase likely interacts primarily with the material (CCSC/CCCS) via its −NH_2_ groups, while the material may interact via its −OH and −NH_2_ groups. Due to the hydrophilic nature of the material, besides electrostatic interactions, hydrophilic interactions may also be significant. Additionally, π–π interactions involving the styrene ring may occur, as styrene was present in the material’s composition in a polystyrene-to-chitosan ratio of 1:3.

Based on these considerations and the data presented in Figure 4a, the binding sites on the protein–substrate complex were ranked, prioritizing positively charged residues (primary; H, R, K, N and Q) and second, residues containing aromatic rings (secondary residues; F and Y). The analysis revealed that the preferred residues on the enzyme that come into contact with the substrate (for binding) are located at sites 1, 2, and 3, with site 3 having the largest number of preferred primary residues. A comprehensive examination of binding sites (1 to 5) is provided in Table S1.

**Figure 4:**
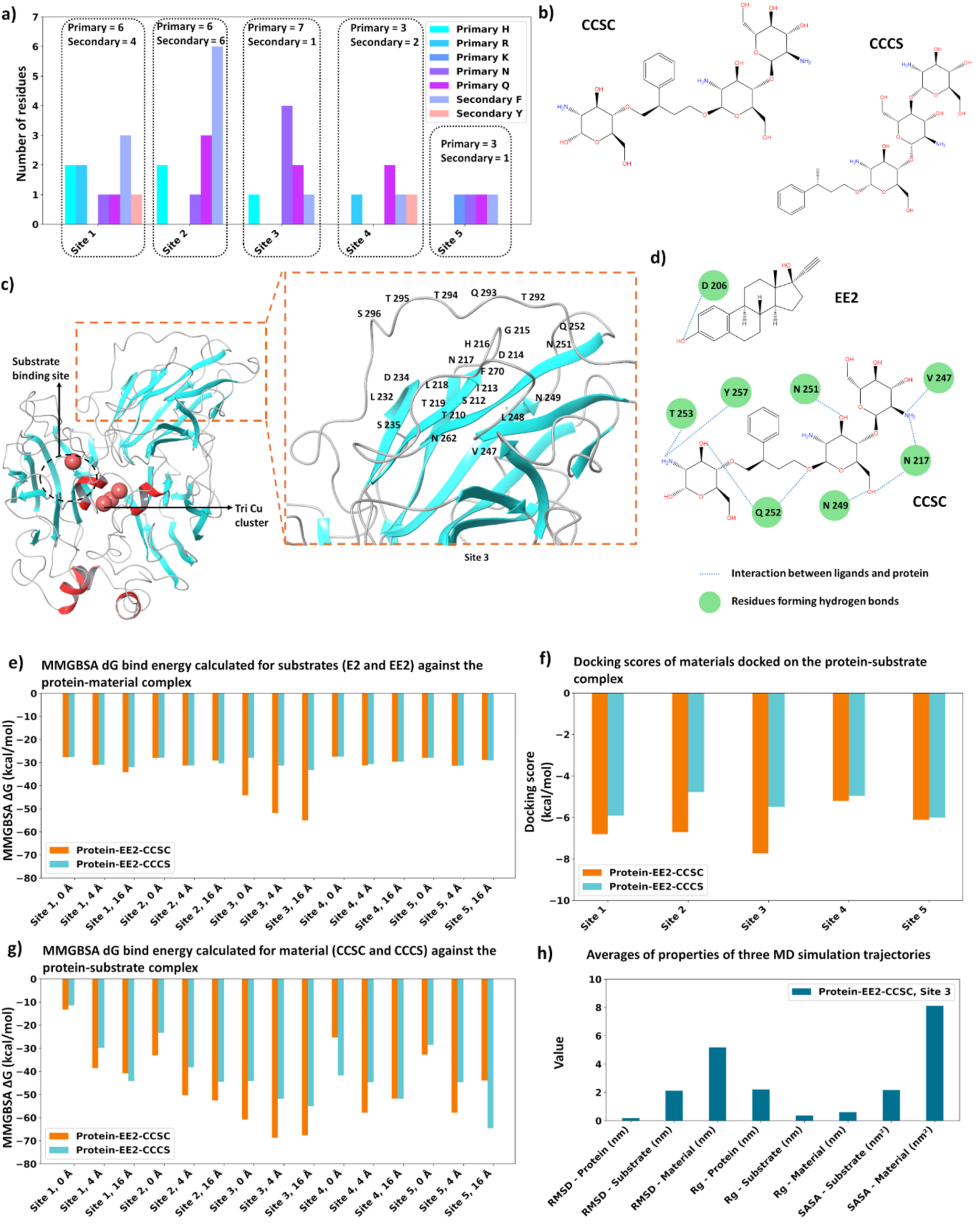
Computational analysis of binding features of the material (CCSC and CCCS) and substrate (EE2). (a) The composition of five potential binding sites on laccase (excluding substrate site) represented in terms of primary (H, R, K, N and Q) and secondary residues (F and Y) preferrable for material binding. (b) Material combinations used for this study in 1:3 for polystyrene (S)-to-chitosan (C) named: CCSC and CCCS. (c) *T. versicolor* laccase structure (PDB code 1GYC), showing the substrate binding site near T1 Cu and the probable site (site 3) for material. (d) Residues interacting with EE2 (at T1 site) and CCSC (at site 3). Residues forming hydrogen bonds are shown in green. (e) MMGBSA Δ*G* (change in Gibbs free energy) binding energy calculated for substrates against the protein-material complex. The sites 1 to 5 represent material binding sites, while the substrates bind to T1 site. (f) Docking scores of materials docked onto the protein–substrate complex. (g) MMGBSA ΔG binding energy calculated for materials (CCSC and CCCS) against the protein–substrate complex. (h) Averages of key properties from three MD simulations for laccase-EE2-CCSC complexes when material was bound to site 3: RMSD (root-mean-square deviation, in nm), *R*_g_ (radius of gyration, in nm), and SASA (solvent-accessible surface area, in nm^2^) for the protein, substrates (EE2), and the material CCSC.

EE2 (possible substrate) was docked at the substrate-binding site (Table S2), while the carrier materials (Figure 4b) were docked at sites 1 through 5. Interestingly, EE2 showed better binding affinity to laccase when the material was bound to site 3 and the material unit was CCSC (Figure 4e). When CCCS was bound to laccase, EE2 exhibited a lower binding affinity to laccase for all five sites. This suggests that site 3 may be the optimal binding site for the material combination CCSC. For confirmation, we computed the docking score and MMGBSA affinities between the material units (CCSC and CCCS) and the laccase in the presence of EE2 substrate for all five binding sites (Figure 4f and g). The MMGBSA binding affinities had previously been found to correlate with the experimental binding at low flexibilities of the binding site, i.e., when approximately zero flexibility was applied. Therefore, we placed emphasis on the MMGBSA scores for 0 Å flexibility. In line with our prior observations, the optimal docking scores and binding affinities were obtained for site 3 in the presence of EE2 substrate. This indicates that this may be the likely binding site for the material and the combination CCSC may bind better than CCCS. The binding analysis showed that site 3 had a notably higher affinity for the material, a feature particularly evident when the protein–substrate complex was bound to CCSC (Figure 4e, f, and g). Furthermore, the lower flexibility (0 Å) of the protein residues applied in MMGBSA helps to maintain configurations close to the crystal structure, resulting in increased binding affinity. Site 3 was identified as the most probable binding site for the material, and the substrate binds to the T1 Cu site (Figure 4c). EE2 forms a hydrogen bond with D 206. The material CCSC interacts with laccase site 3 predominantly via hydrogen bonding, involving residues N217, V247, N249, N251, Q252, T253, and Y257 (Figure 4d). Considering binding site-specific characteristics, docking scores and binding affinity analysis, we further performed MD simulations on the protein–substrate–material complex with CCSC positioned at site 3 for the protein–EE2–CCSC complex (Figures 4h and S1). To analyze variations in conformational flexibility, we calculated the root-mean-square deviation (RMSD, nm), radius of gyration (Rg, nm), and solvent-accessible surface area (SASA, nm^2^) for the protein, ligands, and material (CCSC). Stable trajectories for RMSD, Rg and SASA (Figure 4h and S1) were obtained for the protein–EE2–CCSC complex. The substrate (EE2) remained in the T1 binding pocket for a shorter time and exhibited high dynamics. The material (CCSC) exhibited conformational flexibility at site 3 (Figures 4h and S1), suggestive of its binding to the protein in multiple conformations.

### 3.4 Application of the PS-Chitosan+Laccase System for the Removal of Estrogen

A key goal of the research was to evaluate the effectiveness of free and immobilized laccase in the removal of 17α-ethinylestradiol (EE2), a hormonally active compound, from model and real wastewater, and to investigate the persistence of the immobilized form of the enzyme with repeated use. Laccase has previously been shown to have significant potential for bioremediation processes, enabling the oxidation of a wide range of organic contaminants, including pharmaceuticals and endocrine disruptors, resulting in conversion of the harmful substances into less toxic compounds.

Figure 5a,b shows the results for EE2 removal as a function of time from the model and real wastewater, comparing the efficiencies of free and immobilized laccase. In the case of the model wastewater (Figure 5a), it was observed that the immobilized laccase had a slightly higher efficiency over the whole process time. After 10 h, 70% degradation was observed, while the efficiency of free laccase was just over 60% over the same period. As the reaction time increased, the differences became more pronounced, finally reaching a degradation efficiency of almost 90% for immobilized enzyme and 78% for free laccase after 24 h. The greater efficiency of the immobilized laccase can be attributed to several factors, including improved enzyme stability, protection from denaturation, and prolonged catalytic activity due to restricted conformational changes. These advantages contribute to the observed higher EE2 degradation efficiency, particularly over extended reaction times. The results for the real effluent (Figure 5b) show more significant differences in the efficiencies of the two systems. Immobilized laccase had a higher efficiency over the whole time range, reaching a level of 45% after 24 h. In the case of the free enzyme, the efficiency was slightly lower, reaching only 29% after 24 h. The difference in the efficiency of the two forms of enzyme between the model and real wastewater is due to the presence of additional substances in the real wastewater that may act as inhibitors of free laccase activity. The increased stability of the immobilized enzyme and its resistance to the negative effects of contaminants make this form of enzyme more efficient in the real wastewater environment. Figure 5c shows the results of an assessment of the sustainability and reusability of the biocatalytic systems. A gradual decrease in relative enzymatic activity was observed over five successive cycles for both synthetic and real wastewater. Although the system exhibited higher stability in synthetic wastewater, in both cases the activity declined progressively with each reuse cycle. By the fifth cycle, the relative activity had decreased by approximately 40–50% compared with the initial value, indicating partial loss of catalytic performance. This reduction may be attributed to factors such as enzyme leaching, fouling, or conformational changes of the immobilized enzyme, with more pronounced effects observed in the complex model wastewater matrix.

**Figure 5:**
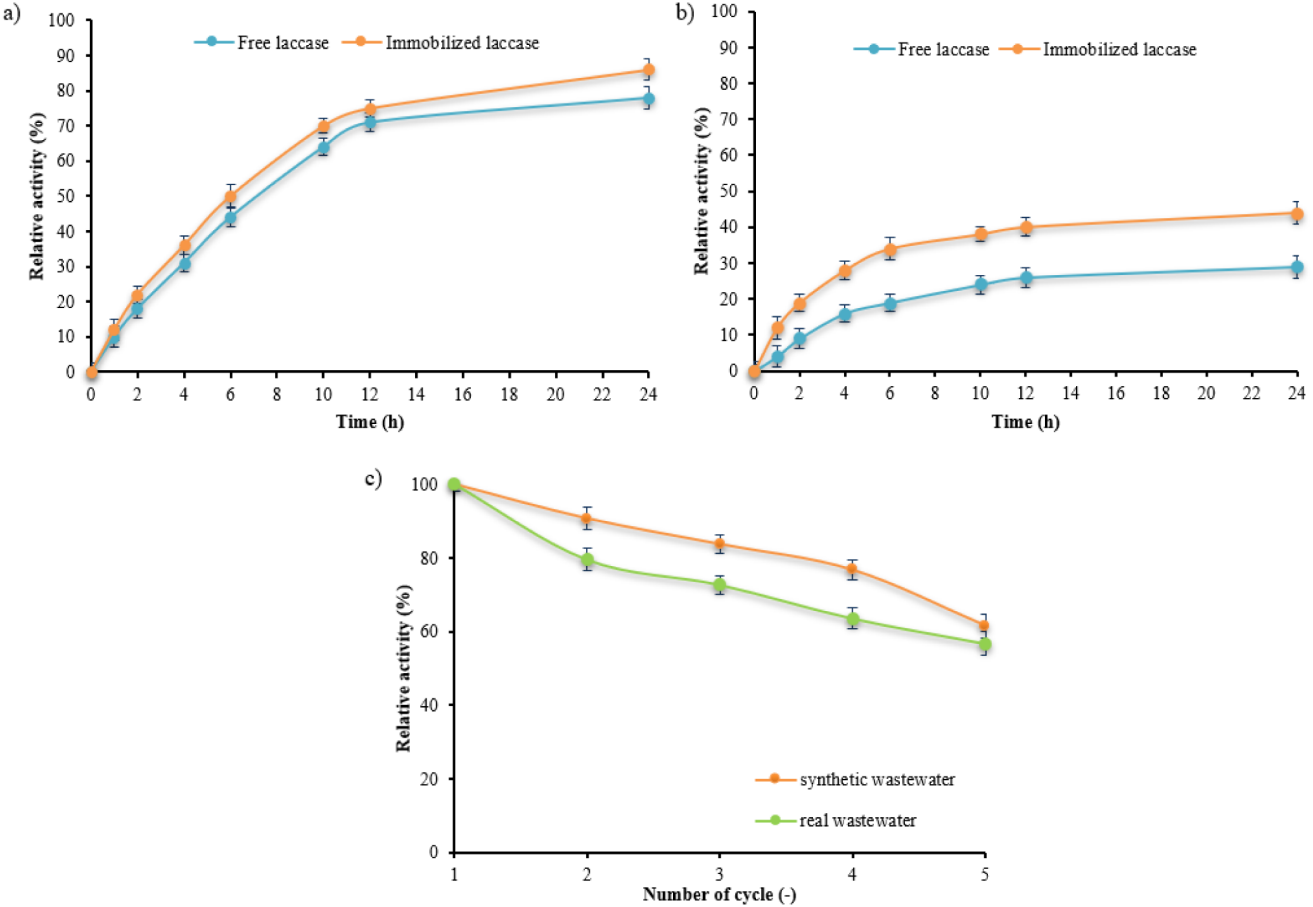
Removal of EE2 from synthetic wastewater (a) and from real wastewater (b) and reusability of the PS-chitosan+laccase system in synthetic wastewater and real wastewater (c), where “absolute” activity was lower at time 0.

The toxicity assessment using the *Artemia salina* bioassay revealed a 70% decrease in acute toxicity following the degradation of EE2. In comparison to the initial EE2 solution, samples obtained after the biological treatment induced markedly lower mortality of *A. salina* larvae, indicating that the transformation process led to less toxic metabolites. This reduction in toxicity suggests that the applied biological process resulted not only in the degradation of the parent compound but also in an overall detoxification of the system. Although *Artemia salina* represents a nontarget organism and provides a general measure of acute toxicity, the observed decrease in mortality confirms that no highly toxic byproducts were formed during EE2 degradation. Consequently, these results strengthen the conclusion that the investigated process exhibits significant biological remediation potential.

### 3.5 EE2 Biodegradation and Examination of Potential Degradation Pathways

Analysis of the process of degradation of 17α-ethinylestradiol reveals several complex biochemical transformations leading to the formation of different classes of metabolites. A key aspect of an ideal degradation process is mineralization, which represents the final breakdown of the compound into inorganic molecules such as carbon dioxide, water, and other simple elements. The occurrence of mineralization is a strong indicator of the complete removal of micropollutants from the environment, reducing their endocrine-disrupting potential. This final step is achieved by the combined action of enzymatic oxidation, hydrolysis, and ring cleavage, ultimately leading to the complete breakdown of the steroid structure.^51^ The HPLC-MS analysis confirms the existence of similar multistep EE2 conversion pathways, featuring oxidation to 17-keto forms, multiple hydroxylations of the A ring, possible isomeric transformations and tautomerism, partial or complete loss of the ethyl group followed by potential further degradation leading to steroid ring opening, as well as mineralization, as illustrated in Figure 6.

**Figure 6:**
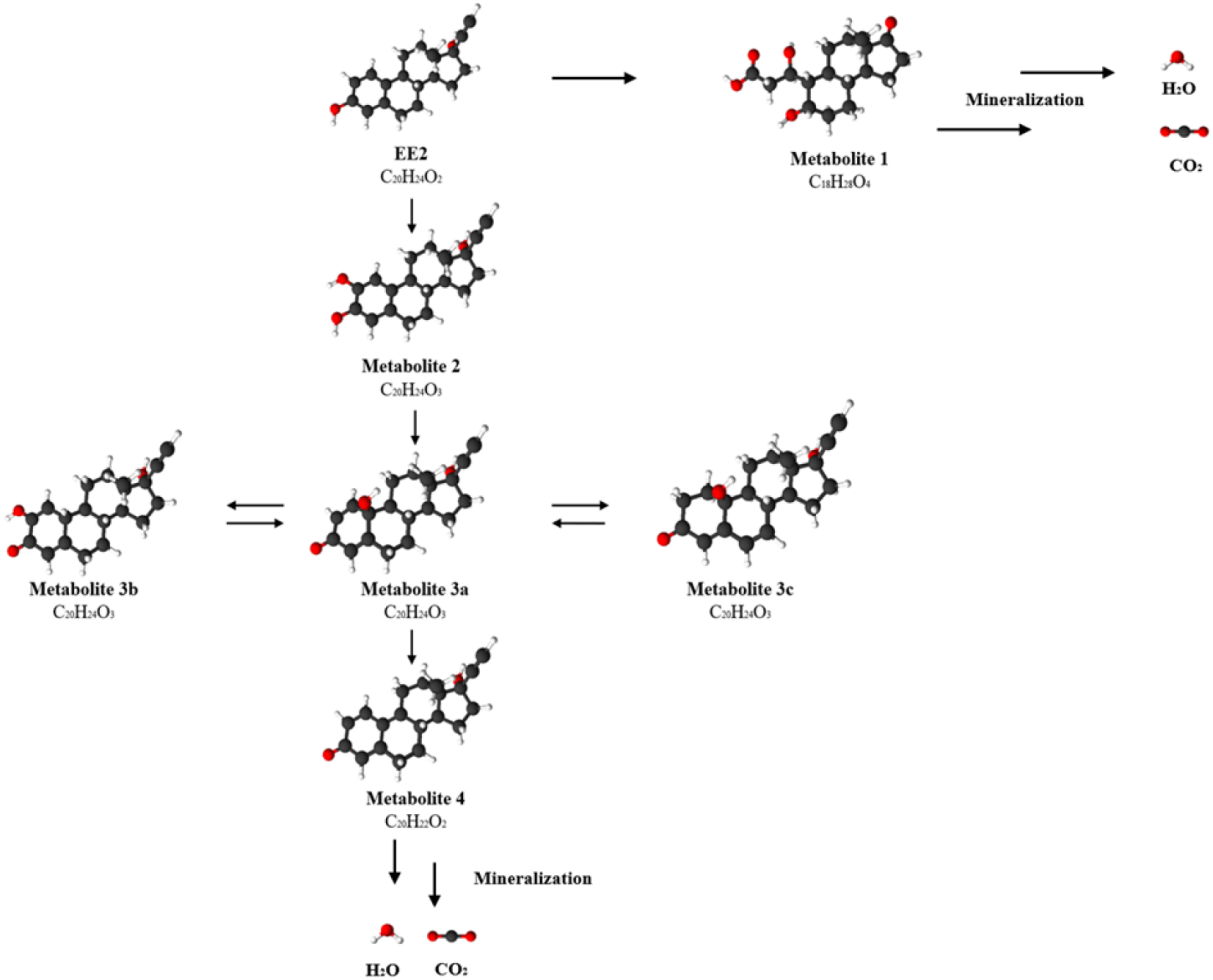
Possible degradation pathway of EE2 catalyzed by PS-chitosan immobilized laccase form *Trametes versicolor*.

The initial step is the oxidation of EE2 to a metabolite with the molecular formula C_20_H_24_O_3_ (Metabolite 2), which is accompanied by the introduction of an additional functional group while maintaining the original 20-carbon skeleton. Subsequently, through further isomerization reactions, Metabolite 2 is transformed into several isomeric forms (designated as 3a, 3b, and 3c), which despite having an identical molecular formula, differ in the distribution of hydroxyl and carbonyl groups - a phenomenon attributable to keto–enol tautomerism or bond rearrangement within the steroid ring system. The subsequent step involves dehydration or dehydrogenation, leading to the formation of Metabolite 4 (C_20_H_22_O_2_), characterized by a reduction in the number of hydrogen and oxygen atoms, which suggests the formation of additional double bonds in the structure. In parallel with these pathways, an alternative degradation mechanism occurs in which EE2 undergoes fragmentation, resulting in the formation of Metabolite 1 (C_18_H_28_O_4_). In this pathway, the loss of two carbon atoms indicates a disruption of steroid ring continuity and is accompanied by intensified oxidative reactions, leading to an increased number of functional groups such as hydroxyl, carbonyl, or acidic groups. It should be highlighted that in the final HPLC results no EE2 was detected, which confirms previous data on the removal rates achieved. The degradation of EE2 was further confirmed by the analysis of total organic carbon (TOC), which also provided an important insight for analysis of the mechanism. The TOC results revealed a significant decrease in carbon concentration from 9.10 g/L to 4.3 g/L after 24 h of incubation in the sample containing EE2 and laccase. The observed 52.75% reduction in TOC indicates an intensive degradation of organic compounds, suggesting the efficiency of enzymatic transformation of the substrate under experimental conditions. This reduction is due to the catalytic activity of laccase, which catalyzes the oxidation of aromatic compounds, including EE2, leading to their conversion into intermediate products with altered chemical structures. This process is aimed at achieving the most complete possible mineralization of organic substances; however, the results indicate that degradation products remain in the sample after 24 h, which may suggest the presence of stable metabolites or incomplete substrate transformation. This is further confirmed by the HPLC results, which showed traces of EE2 metabolites in the mixture after 24 h’ treatment. It is also possible that oxidation results in the formation of more reactive products, which, over time, may undergo further biodegradation or polymerization. The observed decrease in TOC suggests that enzymatic degradation of EE2 proceeds effectively, although it does not yet lead to the complete elimination of all organic reaction products.

The final partial mineralization, or the decomposition of a compound into products such as carbon dioxide, water, and mineral salts, is also an important aspect of these transformations. This process is crucial from a bioremediation point of view, as microorganisms capable of degrading steroids enable the elimination of biologically active compounds from the environment. HPLC analysis confirms the presence of these metabolites, with their diverse spatial structures influencing their physicochemical properties, such as polarity and retention time, thereby enabling effective separation and identification of the individual degradation products. The overall depiction of the EE2 degradation pathway illustrates the complexity of the transformation processes, which include oxidative, isomerization, and dehydration reactions, and thus plays an important role in assessing the compound’s persistence and its potential environmental impact, up to the complete mineralization of the original molecule. Further analysis of the composition of the resulting metabolites is necessary to determine the extent of their degradation and their potential environmental impact.

## 4 Conclusion

The FTIR, CLSM, zeta potential, and SEM analyses have provided comprehensive insights into the process of laccase immobilization on the PS-chitosan carrier, confirming the effectiveness of enzyme binding as well as the chemical and morphological modifications of the material. FTIR spectra revealed the formation of new covalent bonds between functional groups of chitosan and amino acids of the enzyme, indicating permanent immobilization. CLSM observations confirmed the presence of laccase on the support, while changes in zeta potential demonstrated modifications in the electrostatic properties of the material. SEM imaging showed significant morphological changes in PS-chitosan after immobilization, including the presence of enzyme deposits and increased irregularity of fiber structures. Stabilization of the enzyme on the PS-chitosan carrier significantly enhanced its resistance to pH and temperature variations and extended the duration of its activity compared with the free form. Immobilization led to a reduction in the enzyme inactivation rate and a shift in optimal activity toward more favorable operating conditions. The possible binding site (site 3) for the material on laccase was identified, based on the composition of the site residues. The studied substrate (EE2) consistently showed better binding affinity when the material was bound to site 3 than in the case of other sites, and also when the material combination was CCSC. The material combination CCSC was observed to bind better to laccase than CCCS and to site 3; the data thus suggest that it may be possible to target the design of the PS-chitosan material for enhanced binding. When compared with previously reported immobilization systems, such as chitosan-alginate-Cu^2+^ composite hydrogels that improve enzyme stability through ionic cross-linking, MOF ZIF-8 systems with coimmobilized mediators offering high catalytic efficiency via enhanced electron transfer, and electrospun chitosan nanofibers providing high surface area and mass transfer, the PS-chitosan carrier represents a balanced alternative combining effective covalent binding, mechanical robustness, and favorable environmental compatibility. Unlike hydrogel systems that may suffer from diffusional limitations or MOF-based platforms associated with complex synthesis and potential metal leaching, the PS-chitosan support enables stable enzyme immobilization while maintaining operational simplicity and reusability.^15,52,53^ The obtained immobilized system, due to its high enzymatic activity and stability across a wide range of environmental conditions, holds potential for applications in pollutant biodegradation, biosensor production, and other biocatalytic processes. The results confirm the effectiveness of immobilization as a strategy for improving the properties of enzymes and enhancing their applicability in industrial processes. Moreover, the PS-chitosan support is composed of renewable and low-toxicity biopolymers, making it compatible with sustainable material engineering strategies. Chitosan is derived from natural biomass, is biodegradable and exhibits low environmental persistence, while the carrier can be regenerated and reused over multiple catalytic cycles, reducing material consumption and waste generation. The immobilized system therefore aligns with principles of green biocatalysis and offers advantages in terms of resource efficiency, scalability, and reduced environmental footprint, supporting its potential implementation in environmentally relevant remediation processes.

## Supplementary Material



## Notes

Figure S1: properties of protein, material (CCSC), and substrate (E2 and EE2) properties: (a) root mean square deviation (RMSD) values, (b) radius of gyration (Rg), and (c) solvent accessible surface area (SASA); the first two columns show protein properties in the protein-E2-CCSC and protein-EE2-CCSC systems, respectively; the third and fourth columns represent material (CCSC) properties in the protein-E2-CCSC system; the fifth and sixth columns correspond to substrate properties: E2 in protein-E2-CCSC and EE2 in protein-EE2-CCSC; Table S1: detailed analysis of binding sites (1 to 5); Table S2: docking scores, when substrate is docked on protein (PDF)
