# Supplementary material for: Bridging Simulation and Sustainability: Laccase Immobilization on Bio-Polymeric Hybrids for Degradation of 17α-Ethinylestradiol in Water Systems
Source: ACS Omega. 2026 Apr 20;11(17):25406–20. doi: 10.1021/acsomega.5c13237 (PMC13150622; doi:10.1021/acsomega.5c13237)
Supplement: Supplementary file 1 [file ao5c13237_si_001.pdf]

Bridging Simulation and Sustainability: Laccase Immobilization on Bio-Polymeric Hybrids for Degradation of 17α-Ethinylestradiol in Water Systems

Agnieszka Rybarczyk[a], Pranchal Shrivastava[b], Rukmankesh Mehra[b,c], Teofil Jesionowski[a], Anne S. Meyer[d], Jakub Zdarta[a*]

[a] Institute of Chemical Technology and Engineering, Faculty of Chemical Technology, Poznan University of Technology, Berdychowo 4, PL-60965 Poznan, Poland
e-mail: jakub.zdarta@put.poznan.pl

[b] Department of Chemistry, Indian Institute of Technology Bhilai, Durg 491002 Chhattisgarh,India

[c] Department of Bioscience and Biomedical Engineering, Indian Institute of Technology Bhilai, Durg 491002 Chhattisgarh, India

[d] Department of Biotechnology and Biomedicine, DTU Bioengineering, Technical University of Denmark, Soltofts Plads 227, Kgs Lyngby, DK 2800, Denmark

Supplementary data

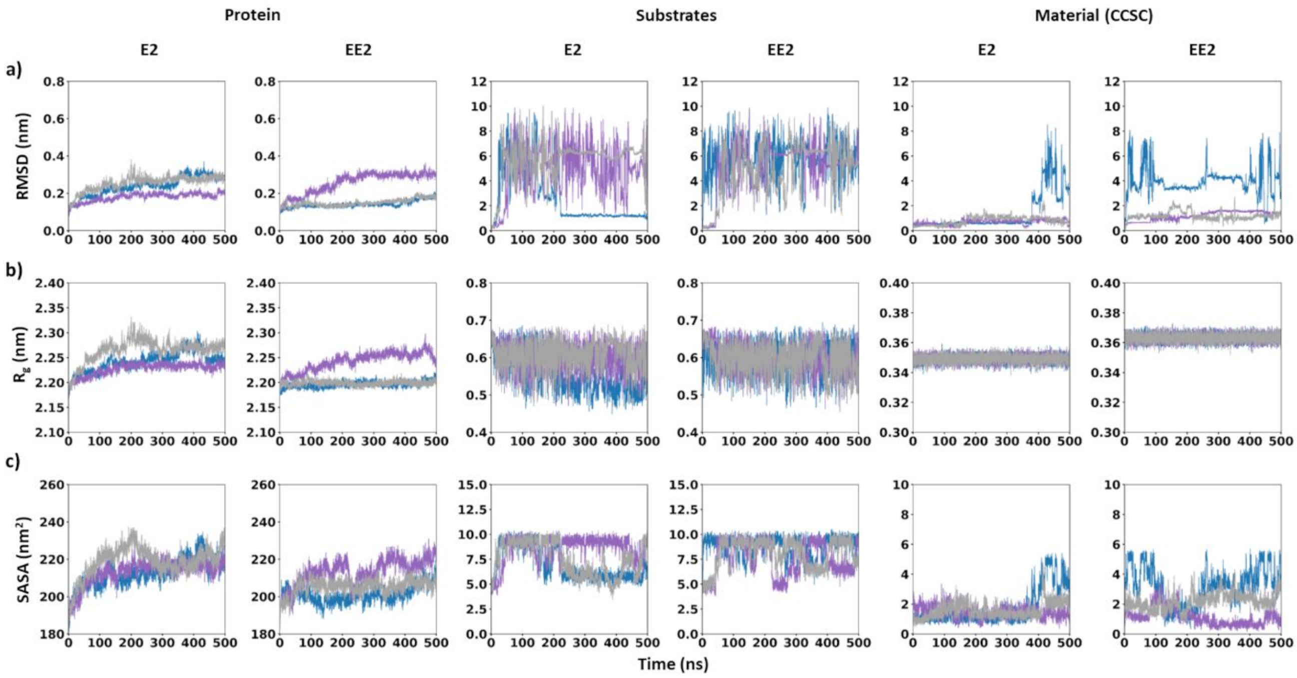

Figure S1. Properties of protein, material (CCSC) and substrate (E2 and EE2) properties. (a) Root mean square deviation (RMSD) values. (b) Radius of gyration (Rg). (c) Solvent accessible surface area (SASA). The first two columns show protein properties in the Protein-E2-CCSC and Protein-EE2-CCSC systems, respectively. The third and fourth columns represent material (CCSC) properties in the Protein-E2-CCSC system. The fifth and sixth columns correspond to substrate properties: E2 in Protein-E2-CCSC and EE2 in Protein-EE2-CCSC.

Table S1. Detailed analysis of binding sites (1 to 5).

**Site-1**		**Site-2**		**Site-3**		**Site-4**		**Site-5**	
**Residue number**	Amino acid	Residue number	Amino acid	Residue number	Amino acid	Residue number	Amino acid	Residue number	Amino Acid
**55**	H	68	F	210	T	235	S	40	K
**58**	L	69	F	212	S	236	I	101	D
**79**	P	70	Q	213	I	237	Q	102	Q
**80**	A	71	A	214	D	239	F	103	A
**111**	H	73	T	215	G	242	Q	106	F
**112**	L	97	F	216	H	244	Y	128	D
**113**	S	98	H	217	N	299	P	131	D
**114**	T	99	V	218	L	300	L	132	P
**156**	A	100	P	219	T	301	I	224	V
**157**	R	101	D	232	L	302	E	225	G
**159**	G	102	Q	234	D	303	T	226	I
**160**	P	311	M	235	S	409	S	227	N
**161**	R	312	P	247	V	410	A	307	P
**163**	P	314	P	248	L	411	G	308	L
**336**	N	401	G	249	N	423	R	309	A
**344**	F	402	H	251	N	426	V	311	M
**345**	T	403	A	252	Q	427	S		
**346**	P	404	F	262	N				
**450**	F	422	F	270	F				
**457**	F	441	F	292	T				
**459**	L	442	Q	293	Q				
**460**	E	444	D	294	T				
**461**	A	445	N	295	T				
**462**	G			296	S				
**491**	Y								
**499**	Q								

Table S2. Docking scores, when substrate is docked on protein.

**Substrate**	**E2**	**EE2**
**Docking score**	-4.48	-4.16
